# Association between breastfeeding and new mothers’ sleep: a unique Australian time use study

**DOI:** 10.1186/s13006-020-00347-z

**Published:** 2021-01-06

**Authors:** Julie P. Smith, Robert I. Forrester

**Affiliations:** 1 Research School of Population Health, College of Health and Medicine, The Australian National University, Canberra, Australia; 2 Statistical Consulting Unit, The Australian National University, Canberra, Australia

**Keywords:** Maternal time use, Sleep, Work, Breastfeeding, Lactation, Breastmilk

## Abstract

**Background:**

Infant sleep is of great interest to new parents. There is ongoing debate about whether infants fed with breastmilk substitutes sleep longer than those exclusively or partially breastfed, but what does this mean for the mother? What expectations are realistic for mothers desiring to exclusively breastfeed as recommended by health authorities?

There are both biological and social influences on infant and maternal sleep. More accurate information on average maternal sleep hours for diverse feeding practices may help guide realistic expectations and better outcomes for mothers, infants and families.

**Methods:**

Using a unique time use dataset purposefully designed to study the time use of new mothers, this study investigated whether the weekly duration of maternal sleep, sleep disturbance, unpaid housework, and free time activities differed by detailed feeding method. The study collected 24/7 time use data from 156 mothers of infants aged 3, 6 and/or 9 months between April 2005 and April 2006, recruited via mother’s groups, infant health clinics, and childcare services throughout Australia. Sociodemographic and feeding status data were collected by questionnaire. Statistical analysis used linear mixed modelling and residual maximum likelihood analysis to compare effects of different infant feeding practices on maternal time use.

**Results:**

There were no significant differences in time spent asleep between lactating and non lactating mothers, though lactating mothers had more time awake at night. Lactating mothers spent more time (8.5 h weekly) in childcaring activity (*p* = 0.007), and in employment (2.7 vs. 1.2 h, *p* < 0.01), but there were no significant differences in free time. Those not breastfeeding spent more time in unpaid domestic work.

Exclusive breastfeeding was associated with reduced maternal sleep hours (average 7.08 h daily). Again, free time did not differ significantly between feeding groups. Exclusively breastfeeding mothers experienced reduced sleep hours, but maintained comparable leisure time to other mothers by allocating their time differently. Domestic work hours differed, interacting in complex ways with infant age and feeding practice.

**Conclusions:**

Optimal breastfeeding may require realistic maternal sleep expectations and equitable sharing of paid and unpaid work burdens with other household members in the months after the birth of an infant.

**Supplementary Information:**

The online version contains supplementary material available at 10.1186/s13006-020-00347-z.

## Background

Sleep hours of infants are an issue of great interest among new parents, and are investigated in many clinical sleep studies [1]. There is also ongoing debate about whether infants fed with breastmilk substitutes sleep longer than those exclusively or partially breastfed [2–6], but what does this mean for the mother? What expectations are realistic for mothers desiring to exclusively breastfeed as recommended by health authorities? Concerns about infant sleep arise from possible associations with later developmental or health problems, but also arise due to links with maternal anxiety or postnatal depression.

Research in this area is hindered by lack of suitable time use and infant feeding data, and by the complex interrelationship between maternal and child sleep. Numerous studies examine infant sleep, and some consider infant feeding, but most studies in this area focus on infant, not maternal, sleep hours. Most also fail to consider how mothers’ sleep and other free time might adapt to demands of ‘night-time parenting’ within the context of their other daily activities.

A better understanding of infant sleep patterns can benefit parents by informing them of what sleep patterns may emerge in their newborn and when more mature sleep development may be expected, and may also help health professional and childcare advisors in counselling parents and guiding realistic parental expectations. This is especially important in the light of growing evidence that interventions to promote infant sleep can have unintended adverse consequences including increased maternal anxiety or premature weaning from breastfeeding [7].

Breastfeeding is well established as an important underpinning of lifelong maternal and child health, as well as supporting child development. Breastfeeding exclusively for six months with continued breastfeeding to 2 years and beyond is recommended by health authorities, for promoting both child and maternal health and wellbeing [8–19].

What should mothers who intend to exclusively breastfeed realistically expect about sleep, for themselves, and for their infant at various ages, and what strategies help families accommodate and adjust to night-time parenting needs of infants?

There are both biological and social influences on infant and maternal sleep, and the existing research in this area is diverse in methodologies and findings [1], as discussed in more detail below. Mothers’ sleep expectations may conform to prevailing social norms for adult sleep, which can be unrealistic for caregivers of newborns. Maintaining exclusive breastfeeding could require mothers to allocate their time differently to mothers who introduce breastmilk substitutes or complementary foods before 6 months, for example, feeding frequently and during the night in order to build an adequate milk supply. More accurate information on average maternal sleep hours for diverse feeding practices may help guide realistic expectations and better outcomes for mothers, infants and families.

### Infant sleep and suckling

Maternal sleep can be anticipated to be highly influenced by their infants’ sleep. Numerous researchers have examined infant sleep, including measuring how many hours children sleep at different ages. A recent systematic review indicates that average 24-h sleep duration among 3 month old infants is 12–18 h, changing only slightly with age to 12.1–14.2 h at 6 months, and 11.3–13.9 h at 9 months [1]. Average infant sleep duration is influenced by cultural and social factors [20]. Some studies also suggest infant sleep hours are affected by feeding method [2–6], (although very few studies compare exclusively breastfed infants, and those partially or fully fed with breastmilk substitutes including solid food). Variation of sleep hours by feeding method is consistent with biological as well as social explanations. Components of breastmilk may influence sleep patterns of infants [1, 21], and feeding rhythms also interact in complex ways with development of more mature diurnal sleep rhythms in newborns [22]. For example breastmilk contains hormones such as leptin and melatonin influencing appetite and sleep respectively, and milk fat content is lower during the night and morning feedings [23]. It has been proposed that diurnal sleep rhythms of infants consolidate earlier than feeding rhythms [22].

### Maternal sleep

Much of the literature on the topic of infant feeding and sleep focusses on sleep of newborn infants. The extent of actual or perceived disturbance of the mothers’ daily activities, such as disruption of maternal sleep, or time available for necessary personal care, leisure or work activities is investigated in fewer studies. Such investigations have tended to focus on implications of reported infant sleep problems for maternal post-natal depression risk [24].

Just as lactation biology influences infant sleep, maternal sleep can also be expected to vary by feeding method. In a US study, exclusively breastfeeding women averaged 30 min more nocturnal sleep than women who used formula at night, but measures of sleep fragmentation did not differ [25]. Lactation hormones such as prolactin help mothers adapt to the stresses of caring for an infant, including broken sleep [13, 26, 27]. Lactation hormones also influence maternal nurturing behavior and desire for proximity to the infant, in animals as well as humans. For example, time use research on new mothers in Australia suggests differences in the amount of time they spent holding and soothing breastfed compared to non-breastfed infants and this was related to the degree of breastfeeding [28, 29].

### Cultural and social factors

Time use is related in socially as well as biologically complex ways to infant feeding. How sleep is perceived rather than the objective duration of sleep may better indicate its effect on maternal well-being [30, 31]. Hence, understanding of how the sleep hours of mother-infant dyads relate to infant feeding needs to be placed within the broader context of how mothers of newborns may prefer or choose to allocate their time between sleep, work (paid or unpaid) and leisure.

Time is increasingly recognised to be a resource needed for good health [32], including for nurturing care and breastfeeding of infants [28, 33]. Who has access to (spare) time resources has been shown in a recent study to be significantly shaped by both socioeconomic class and gender [32]. Being pressed for time (‘rushed’) is linked to being a woman, a sole parent, or disabled, and is also associated with lack of job control and work family conflicts [32]. Thus, access to maternal sleep may depend in part on cultural and social determinants of monetary and time resources available to the mother for infant care. Medical anthropology and ethnographic studies suggest that maternal care activities such as breastfeeding and introduction of other nutrition are adaptive to resource pressures on mothers and their families [34–36]. Time available for optimal infant care including exclusive breastfeeding – which is time intensive [33] - may depend on whether mothers need to conserve maternal resources. For example, undernourished or time-pressed mothers may end exclusive breastfeeding so they can allocate their time to securing food or other essential resources.

A resource-economising perspective points to a previously unexamined mechanism by which mothers adapt to nighttime parenting of infants. This is to reallocate time, whether from daytime leisure or work commitments, such as employment, childcaring or domestic work, to sleeping or napping. Some mothers will have more capacity to do this and maintain their preferred amount of free time activities, or address needs for sleep or personal care time, if they are resourced by others, including other family members, extended kinship groups or the wider community [37, 38]. This can take the form of help from others in dealing with domestic commitments such as housework, shopping, or childcare. Alternatively, it might mean lower maternal hours in income earning activities, such as that afforded by maternity leave. As well as lactation biology affecting infant and maternal sleep behaviours, research has shown cultural differences in infant sleep [20].

The above suggests a significant role for cultural, social and economic factors in influencing infant and maternal sleep and its relation to infant feeding practices. The extent to which caring for an infant interferes with maternal activities such as free time for recreation and socialising, and on work commitments, paid and unpaid, is largely undocumented, but is a measure that may provide new understanding of how sleep duration of the mother-infant dyad affects the health and well-being of both. Sociological studies highlight the gendered nature of sleep disruption among parents of young children [39]. Recent studies show that an important outcome associated with improved access to maternity leave in European countries is better maternal mental health [40, 41].

A novel way to explore the complexities and dynamics of how mothers adapt to the bio-behavioral demands of infant care and to their social context is by investigating whether maternal time spent in key categories of daily activities differs by detailed infant feeding practice. Hence, this study asks how maternal sleep duration relates to infant feeding practices and breastfeeding, and explores how this fits into the broader context of mothers’ daily hours of work and leisure.

## Methods

### Aim

The aim of this study is to identify associations between maternal sleep hours and feeding practices, and explore how maternal sleep hours may relate to mothers’ domestic or other work commitments, and free time for leisure or personal care.

### Design, setting, and participants

Comprehensive time use data on maternal time in the care of infants is rarely collected, particularly in official time use surveys. The Time Use Survey of New Mothers (TUSNM) was a unique nationwide Australian study conducted through the Australian National University (ANU) between April 2005 and April 2006 and specifically designed to address deficiencies in existing time use data collections. All participants gave written informed consent before enrolment (Protocol 2005/51 approved on 10 March 2005 by the ANU Human Research Ethics Committee under the *National Statement on Ethical Conduct in Research Involving Humans* (1999)) [42].

The survey purpose was described to participants as ‘measuring the time it takes to care for a baby’. Recruitment was through national playgroup and breastfeeding support organizations, maternal and child health professional networks, infant health clinics, and childcare centers. Mothers with infants up to age nine months were eligible to participate in the survey and could participate in tracking sessions at time points when the target infant age was three, six and/or nine months.

### Data collection

Data on infant feeding method for the youngest child (the ‘target infant’) during seven days of time use tracking was collected by questionnaire filled out by participant mothers. We excluded participants who recorded time use for less than one 24-h day. Feeding method over the 7 days of tracking was self-reported by participants in the following categories: (A) exclusive breastfeeding; (B) exclusive formula feeding; (C) mixed breastmilk and formula milk – no solids; (D) breastmilk and solids; (E) formula milk and solids; (F) mixed breastmilk and formula milk with solids. Self-report of feeding method was verified by cross checking against individual time use data on feeding activities. Mothers using breast pumps were instructed to record expressing breastmilk as ‘preparing feeds’; these few mothers were categorized as breastfeeding mothers even though feeding this milk might be by someone other than the mother.

Data collected from the mother by questionnaire also included the number of hours and minutes another family member (usually her partner or husband) was caring for, and feeding the infant, and how many hours the infant spent in paid childcare. Fathers were not invited to participate in time use tracking mainly because of ethical concerns to limit response burdens on households. Socio-demographic data were also collected via the written questionnaire.

Participants were asked to track their time use for seven days, 24 h a day, using TimeCorder™ time tracking devices. These were posted to the mothers along with the questionnaire at each tracking time point, that is, within two weeks of the relevant anniversary of the target infant’s birthdate. Participants could record at target infant age three months, six months and/or nine months. Data on the frequency, duration, and time of day of each activity was recorded through participants pressing one of 25 buttons on the device corresponding to their current activity.

### Measuring and categorizing feeding method and time use

An important factor of interest in this study was infant feeding practice. This was measured using two different categorizations. We compared those giving ‘no’ breastfeeding (exclusive formula feeding (feeding category B) & formula milk and solids (E)) with those giving ‘any’ breastfeeding (exclusive breastfeeding (A), mixed breast milk and formula milk – no solids (C), breastmilk and solids (D), mixed breastmilk and formula milk with solids (F)) that is, ‘lactating’ mothers. Maternal hours in the different categories of activities were also compared for the 6 detailed feeding categories noted above (A to F).

TUSNM design and measurement of time use was based on the Australian Time Use Survey (henceforth ‘TUS’), [43]. last conducted by the Australian Bureau of Statistics (ABS) in 2006. The TUS measures main activities in the general population as comprising ‘personal care’; ‘employment’; ‘education’; ‘domestic activities’; ‘purchasing goods and services’; ‘voluntary work and care’; ‘recreation fitness and leisure’; ‘social and community interactions’, and ‘childcare’.

### Topologies of time

**Fig. 1:**
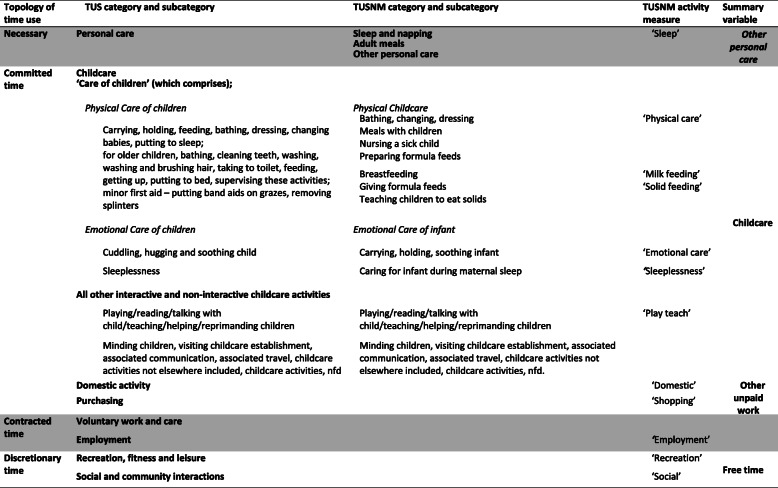
Time use measures and topologies

Most quantitative time use studies categorise the hours/minutes spent according to whether the activity is *contracted* (employment and related travel) or *committed* (caregiving, unpaid or domestic work) or *necessary* (personal care, sleep and eating) activities [44, 45]**.** The balance is considered discretionary or free time, such as for recreation and leisure or social activities. Figure 1 summarises this topology of activity categories, for the TUS and as adapted for this study of maternal time use.

Dependent variables and summary measures were created and categorized to represent *necessary time*, *contracted or committed* time, or ‘*free time’* for mothers of infants aged 3–9 months in line with the topology in Fig. 1.

Time use variables for *necessary* activities are those related to sleeping, eating and other personal care. Corresponding directly to main activity categories in the TUS, the TUSNM included measures of the amount of time spent on “other personal care” (which relates to activities such as bathing or showering). The TUSNM variable labelled ‘sleep’ is defined as maternal time spent “sleeping or napping”. In this study *necessary time* is taken to exclude ‘family meals’, and only includes meals eaten alone by an adult. This is because in families with infants and young children, adults’ time at family meals is more akin to childcare, as children need assistance and supervision with eating, rather than being mainly personal care. Hence, time measured by the TUSNM variable for ‘family meals’ is classified as *committed* time.

The time use of a mother is closely interrelated with commitment to meeting the care needs of her infant. Night-time parenting activities illustrate the interrelationship of *necessary* with *committed* time in this circumstance, and the interrelatedness of the time use activities of the mother-infant dyad. Participants were instructed to record periods of time involving interruptions to maternal sleep (‘sleeplessness including awake while feeding a baby during the night’) as “sleeplessness”. In this dataset, “sleeplessness” is defined as the cumulative time the mother recorded being awake during the night tending to the infant, or otherwise being awake during the night. The ‘sleeplessness’ categorization was intended to provide for night-time infant care activity such as the mother dozing and/or the infant feeding to sleep. Nevertheless, where there was ambiguity about how to classify any such activities throughout the study, mothers were instructed to allocate their time according to how they viewed the main purpose of their activity at that time. Hence, ‘sleeplessness’ could exclude time actually feeding the infant, if the mother viewed “breastfeeding” rather than “sleeping or napping” as the main purpose of her activity at that time. Likewise, nappy changing at night would be categorized under “bathing, changing or dressing” the child, if the mother considered that to be the main purpose of her activity at that time. Such a category of activity is not included in the TUS which is designed for measuring time us of the general population of adults not the distinctive time use activities of mothers of infants. This term was included in the TUSNM to reflect the unique, dyadic characteristics of this population sub group and time use activities of new parents.

“Sleeplessness” is categorized here as *committed* time, being a form of childcare. It is comparable to adults’ commitments to unpaid domestic work, or care of children. Childcare in TUSNM involves the physical or the emotional care of an infant, including feeding as well as soothing, holding, or bathing a child, consistent with TUS.

Reflecting the multitasking nature of infant care, a summary measure of nighttime parenting, combining hours of sleeping or napping, plus periods of disturbed sleep (‘sleeplessness’) was also defined for this study, which overlaps *necessary* and *committed* time. This variable indicates how conflict between using time for necessary maternal sleep activity was reconciled with commitment to maternal care of the infant at night.

The ABS TUS also includes measures of *discretionary* activity, such as recreation and leisure, or social and community interactions. These are similarly measured in TUSNM, as ‘recreation’ and ‘social’ activity time use. To provide further context for analyses of maternal sleep, we report analyses of data on maternal time spent in our summary measure, ‘free time’. ‘Free time’ is constructed as the sum of maternal time spent in “recreation” activity and time spent in “social” activity.

### Statistical analysis

T-tests and chi-square tests were used to compare the socio-demographic characteristics of the two infant feeding groups. Characteristics of participants who provided time tracking data records at one, two or three time points (that is, at infant ages three, six, and/or nine months) were also compared, using the same techniques.

We used a residual maximum likelihood (REML) analysis in GenStat [46] to perform a linear mixed effects analysis of the relationship between infant feeding category, and the specified maternal time use activities, for participants observed at three time points. Data for time use variables were transformed by taking the square root prior to analysis, to stabilize the variance.

A repeated measures approach to statistical analysis was appropriate because at least one but up to three successive time use and sociodemographic data observations were provided by participants when the target infant was aged 3, 6 and/or 9 months, and because the numbers of observations in the cells of the two-way tables were highly variable. The intention of using the REML method is to address the repeated measures and unbalanced nature of the data. REML exploits all available observations for each participant at the three time points, rather than omitting information from participants with missing data, and puts more weight on cells with more observations in a multi-way table.

A REML mixed effect model was preferred over traditional approaches such as repeated measures analysis of variance (ANOVA) as the focus of the study was the effect of infant feeding practice on maternal time use. Mixed effect modelling allowed us to estimate the separate (main) effects of feeding category and infant age, whilst also exploring how the interaction of these two (fixed) factors affected maternal time use.

REML analyses were conducted for the two factors of interest described earlier; ‘detailed feeding category’ (six levels), and breastfeeding (including mixed) versus non-breastfeeding (two levels). The fixed terms in the model were “infant age”, “factor of interest”, and the interaction between these. The interaction checks the two-way table between “infant age” and “factor of interest” to see whether there are significant differences between any pair of means in the table that is not attributable to either the main effects of the fixed terms “infant age” or “factor of interest”. The random terms were “target infant”/ “infant age” (which expands to “target infant” and “infant age” within “target infant”).

The significance of fixed effects (*p* < 0.05) was assessed on the transformed data using Wald statistics and approximate F-statistics. This is appropriate for assessing the significance of both the main effects and the interaction term in the model for fixed effects specified above.

## Results

### Participants

Recruitment generated 185 participants, from an unknown number of contacts. Of 185 mothers giving consent, 162 participated in postnatal time use tracking, and 156 contributed time use and socio-demographic data records. This generated 327 usable data records of maternal time use activities over seven days and nights as inputs to the modelling.

The number of data records/observations exceeds the number of participants because most provided time use tracking data at more than one time point. For example, 31 observations were contributed by mothers who only tracked once, while a further 134 observations were from those who tracked twice. Around half of the 327 observations (*n* = 162) were from those who tracked three times, firstly at three months of infant age, then at six months, then again at nine months.

Table 1 summarizes the dataset presenting information on the age of the target infants at the maternal time use tracking time points, the feeding categories of the target infants at that time point, and tracking categories, describing the number of participants who tracked at one, two or three points. These observations are for 156 individual infants who may be in different feeding groups at different ages, for example, being exclusively breastfed at 3 months, but contributing an observation in the ‘breastfeeding with solids’ feeding group when time use is tracked at 6 or 9 months.

**Table 1:** Summary of observations and data collection: detailed feeding group by age of target infant, and data collection category

Detailed feeding group	Observations by age of target child (n)			Observations (%)
	3 months	6 months	9 months	
A Breastfed only	79	15	1	95 (29.1%)
B Formula only	4	0	1	5 (1.5%)
C Breastfed and formula fed	2	1	0	3 (0.9%)
D Breastfed and solids	1	101	83	185 (56.6%)
E Formula fed and solids	0	11	11	22 (6.7%)
F Breastfed and formula fed and solids	0	6	11	17 (5.2%)
All feeding groups	86 (26.3%)	134 (41.0%)	107 (32.7%)	327 (100%)

Comparison of socio-demographic characteristics for those doing one, two and three time use trackings also showed no statistically significant differences in age of the target child, age of the 2nd youngest child, number of children, mother’s age, mother’s education, or mother’s employment status between mothers who provided data at one, two, or three time points.

The TUSNM sample population had similar characteristics to the Australian population of mothers of infants on most key socio-demographic variables, though participants were more likely to be first-time mothers, and more highly educated. TUSNM also contained a higher prevalence of breastfeeding mothers than would be expected from population based studies of breastfeeding in Australia, such as the Longitudinal Study of Australian Children and the Australian Infant Feeding Survey [47–49].

Maternal age, number of children, the proportion with only one child, and the age of second youngest child were not significantly different between the breastfeeding and non-breastfeeding groups, nor were there significant differences in maternal employment, education levels, and family income.

### Maternal time use – sleep and free time comparisons for combined feeding groups

Table 2 comprises two parts, a and b, and compares the activities of non-breastfeeding/non-lactating mothers (“no breastfeeding”) with breastfeeding/lactating mothers (“any breastfeeding”), and infant ages 3, 6 and 9 months. The table presents back-transformed means. (The predicted means together with back-transformed means are in the Supplementary Tables).

**Table 2:** Maternal weekly hours spent in unpaid childcare and other domestic work, personal care and free time activities, combined feeding groups^(a)^

**a. Activity/Breastfeeding status**	**No breastfeeding**^(b)^ (obs = 27)	**Any breastfeeding**^(b)^ (obs = 300)		***p*-value**
**Necessary time**				
Sleep, adult meals and other personal care time	65.48	63.43		.409
Maternal sleep	58.13	57.41		.085
**Committed time**				
Childcare	39.39	47.89		.007
Sleeplessness	1.63	3.48		.029
Other unpaid work	21.51	17.72		.039
**Free time**	22.26	19.47		.286
Night-time (sleep plus disturbed sleep)	61.20	59.32		.379
**b. Activity/Age of target infant (months)**	**3 months** (obs = 86)	**6 months** (obs = 134)	**9 months** (obs = 107)	***p*-value**
**Necessary time**				
Sleep, adult meals and other personal care time	64.18	65.59	63.58	.186
Sleep	55.79	57.32	55.65	.202
**Committed time**				
Childcare	47.1	42.97	40.66	< 0.001
Sleeplessness	2.98	2.33	2.18	.135
Other unpaid work	17.77	20.31	20.70	.003
**Free time**	21.60	19.86	21.09	.342
Night-time (sleep plus disturbed sleep)	60.25	61.28	59.24	.154

^(a)^ Back- transformed means from residual maximum likelihood analysis, using linear mixed model*.* Note the back-transformed means will be similar, but not be the same as the predicted means for the original data due to the square root transformation and the unbalanced nature of the data. See supplementary table for predicted means and average standard error of difference. ^(b)^ No breastfeeding is exclusive formula feeding (feeding category B) & formula milk and solids (E)). Any breastfeeding is exclusive breastfeeding (feeding category A), mixed breast milk and formula milk – no solids (C), breastmilk and solids (D), mixed breastmilk and formula milk with solids (F))

The first half of the (Table 2a) reports the ‘main effect’ of no breastfeeding with any breastfeeding, on the maternal activities analysed, using the REML method to appropriately weight the data over the different aged target infants. These numbers, in effect, show hours of maternal activity by breastfeeding status, averaged over the whole sample. The second part of the (Table 2b) shows the ‘main effect’ of infant age 3, 6 or 9 months, on maternal activities, for either breastfeeding status (“some” or “no”).

So for example, in Table 2a, the average weekly hours of maternal sleep for a non-breastfeeding mother are 58.13 regardless of the age of the child*.* Testing using the Wald statistics and approximate F statistics did not reveal any statistically signficant interaction between infant age and breastfeeding status.

Similarly, in Table 2b the average weekly hours of maternal sleep for a infant aged 6 months are 57.32 regardless of the breastfeeding status of the child.

Table 2a shows that there were no significant differences in time spent asleep between lactating and non lactating mothers (*p* = .085). Weekly hours of sleeplessness were greater for lactating mothers (*p* = .029). Lactating mothers spent 8.5 h more weekly in childcaring activity (*p* = 0.007). As shown elsewhere [28], average weekly employment hours were higher for lactating mothers (2.7 vs. 1.2 h, *p* < 0.01), as were childcare hours (47.89 vs 39.39, *p* < .01). There were no significant differences in free time between lactating and non lactating mothers. As shown in Table 2a, in comparisons of lactating with non-lactating women, other weekly unpaid work was significantly higher for those not breastfeeding compared to those who were breastfeeding (*p* = .039).

Mothers of older infants did not spend significantly more or less time asleep than mothers of younger infants (Table 2b, *p* = .13). Free time remained at around 20–21 h a week for all ages of infant. Mothers of older infants spent significantly more hours on unpaid housework, around 3 additional hours weekly (*p* = .003), but less on childcare (*p* < .001).

### Maternal time use – sleep and free time comparisons for detailed feeding groups

Table 3 also comprises two parts a and b, and reports predicted means for maternal time spent in various activities. This table is by detailed infant feeding group, and by infant age. The two parts are to be interpreted similarly to Table 2.

**Table 3:** Maternal weekly hours spent in unpaid childcare, personal care and free time activities, detailed feeding groups^(a)^

**a. Maternal weekly hours spent in activity, by detailed feeding group (for all target infant ages)**							
**Activity/Breastfeeding status**	A. Breastfed only (obs = 95)	B. Formula only (obs = 5)	C. Breastfed & formula fed (obs = 3)	D. Breastfed & solids (obs = 185)	E. Formula fed & solids (obs = 22)	F. Breastfed & formula fed & solids (obs = 17)	*p*-value
**Necessary time**							
Sleep, adult meals and other personal care time	58.46	66.24	62.93	66.08	67.70	68.91	.024
Sleep	49.56	58.38	56.54	57.03	60.25	59.04	.004
**Committed time**							
Childcare	49.48	38.79	41.43	47.60	38.09	43.31	.060
Sleeplessness	3.24	1.51	7.24	3.59	1.71	3.17	.222
**Free time**	20.66	19.87	21.10	18.99	22.01	17.01	.719
Night-time parenting (sleep plus sleeplessness)	54.45	60.72	64.56	61.91	63.30	63.92	.008
**b. Maternal weekly hours spent in activity, by age of target infant (for detailed feeding group)**							
Activity	**Age of target infant (months)**				*p*-value		
	3 (obs = 86)	6 (obs = 134)		9 (obs = 107)			
**Necessary time**							
Sleep, adult meals and other personal care time	69.44	64.32		61.43	.006		
Maternal sleep	60.87	55.98		53.52	.006		
**Committed time**							
Childcare	45.30	42.85		40.95	.239		
Sleeplessness	3.94	2.95		2.72	.510		
**Free time**	19.64	19.30		20.80	.445		
Night-time parenting (sleep plus sleeplessness)	66.60	52.85		49.90	.002		

^(a)^ Back- transformed means from residual maximum likelihood analysis, using linear mixed model. Note the back-transformed means will be similar, but not be the same as the predicted means for the original data due to the square root transformation and the unbalanced nature of the data. See supplementary table for predicted means and average standard error of difference

Small cell sizes for formula fed infants (*n* = 3, *n* = 5, *n* = 17), some of whom were also breastfed, suggest the need for caution in interpreting differences, as differences between any particular detailed feeding group categories may or may not be statistically significant. Hence, we discuss results only for comparisons where cell size is large (see Table 1), and mean differences are substantial.

Table 3a shows that unlike for comparisons between combined feeding groups (reported in Table 2), there were significant differences (*p* = .004) in maternal sleep time of up to around 10 h a week between the 6 detailed feeding groups. Those exclusively breastfeeding spent the least time sleeping or napping (averaging 49.56 h weekly, 7.08 h daily). Average hours committed to nighttime parenting, ‘sleeplessness’, were not significantly different between the feeding groups. As in the combined feeding group analysis, there were also no statistically significant differences in mothers’ free time between these detailed feeding groups.

Maternal sleep hours were significantly different by infant age for the detailed feeding groups (Table 3b). Time spent asleep by mothers was significantly less (*p* = .006) if they had older infants. Analysis for detailed feeding groups showed no significant differences in the mothers’ hours of ‘sleeplessness’ for older babies compared to younger babies. However, nighttime parenting hours declined as there were reduced maternal hours of both sleep and sleeplessness as infants got older (*p* = .002). This suggests reduced conflict in time use between ‘necessary’ hours of maternal of sleep and maternal hours ‘committed’ to nighttime parenting as infants matured.

Mothers’ time in childcaring activities, or enjoying free time activities did not change significantly with infant age when analysed for detailed feeding groups.

### Maternal time use – statistical interaction of detailed feeding group with target infant age

Testing using Wald statistics and approximate F-statistics did not reveal statistically significant interactions between target infant age and feeding group on these maternal time use activities for combined feeding groups (Table 2). However, for the detailed feeding groups in Table 3 infant feeding category did interact significantly with age of infant for unpaid work hours. We therefore calculated predicted means for this activity to explore these interactions more fully (Table 4). (The number of observations for each pairing is in Table 1.)

**Table 4:** Relationships between detailed feeding group and maternal sleep outcomes – statistical interaction with target infant age (*P* = .039), maternal weekly hours(^a^}

Feeding group/Age of target infant	3 months	6 months	9 months
A. Breastfed only	16.02	16.84	13.51
B. Formula only	20.69		14.52
C. Breastfed & formula fed	16.08	23.46	
D. Breastfed & solids	9.59	17.88	19.25
E. Formula fed &solids		25.96	22.53
F. Breastfed & formula fed & solids		26.05	17.08

^(a)^ Back- transformed means from residual maximum likelihood analysis, using linear mixed model. Note the back-transformed means will be similar, but not be the same as the predicted means for the original data due to the square root transformation and the unbalanced nature of the data. See supplementary table for predicted means and average standard error of difference

Table 4 presents a more complex picture, allowing a potentially more nuanced interpretation of the results in Tables 2 and 3. Small cell sizes for mothers who were using formula, especially in the early months (see Table 1), emphasise the need for caution in interpreting differences. Differences between any particular detailed feeding group or infant age categories may or may not be statistically significant. As noted earlier, in the combined analysis (Table 2a), unpaid domestic (house)work hours were significantly greater for those that were not breastfeeding, and mothers of older infants did significantly more unpaid housework than mothers of younger (Table 2b). Table 4 showing weekly unpaid work hours for the detailed feeding groups reveals that at three months, those who were exclusively breastfeeding as recommended spent 16.02 h weekly on housework, and at nine months, those still breastfeeding with solids as recommended spent 19.25 h weekly doing housework. Results for other feeding groups are more difficult to compare but the interaction of infant age and detailed feeding group suggest that maternal unpaid work burdens may be relevant for understanding relationships between optimal breastfeeding and maternal sleep outcomes.

## Discussion

This time use study of new mothers addressed questions about whether maternal sleep hours are associated with infant feeding method, and explored how sleep fitted into mothers’ daily work and leisure time for different categories of infant feeding. Using a sample of Australian breastfeeding and non-breastfeeding mothers with broadly similar socioeconomic and demographic characteristics, we used innovative statistical analysis techniques to exploit opportunities offered by time use data records providing week-long (cross sectional and repeated measures) observations of maternal activity at 3, 6 and 9 months of infant age. The first key finding is that there were no significant differences in average hours of sleep when comparing ‘breastfeeding’ with ‘non-breastfeeding’ women, although breastfeeding involved more time being ‘sleepless’ at night.

A second key finding is that maternal free time for recreation and social activities did not differ significantly between feeding groups, despite the varied night-time parenting patterns. Thirdly, this study provides the first analysis of maternal sleep alongside unpaid housework activity. It found that lower hours were spent on unpaid housework by lactating women, who spent more hours instead on providing childcare. We interpret these results as suggesting that mothers adapted their daily work commitments to prioritise some minimum free time, but as infant age increased, mothers reduced childcare hours and spent more time on unpaid domestic work (or employment).

In our analysis by more detailed feeding group, exclusive breastfeeding was associated with lower maternal sleep hours, and surprisingly, the REML analysis showed that maternal sleep hours were significantly lower for those with older infants and more diversified infant feeding practices. This emphasises that improved understanding of how infant sleep and feeding affects women and night-time parenting may require categorising feeding practices in detail, and not dichotomously. Also, the relationship between infant feeding and unpaid domestic work interacted significantly with infant age only in the detailed feeding group analysis. This reflects that maternal time on unpaid childcare and housework also changes in complex ways as infants mature and as feeding practices move away from exclusive breastfeeding after the early months. Our analysis by detailed feeding group showed that mothers who used formula during the first 6 months spent more time on housework than those who did not, but the causal direction is not clear. We note that some cell sizes are not large enough to draw strong inferences between detailed feeding groups.

Current research is focused on infant sleep, and indicates conflicting results comparing infant sleep duration for breastfeeding versus formula feeding. It indicates generally less night waking and longer continued night sleep in formula fed infants, though feeding-related sleep differences are also associated with higher mortality in formula fed infants such as SIDS, and morbidity including wheezing problems [2, 4, 50]. Limitations of existing research include firstly, that many studies do not disaggregate by detailed feeding method, and thus may include formula or mixed fed with partly breastfed infants. The effect on infant sleep of feeding solids has also been specifically investigated, with conflicting results reported [4, 5]. Secondly, some studies do not account for increasing infant age on sleep duration and night waking patterns; many do not examine whether differences in sleep patterns are sustained past 6 months [2, 3, 6]. Differences evident in studies of infant sleep by age point to the importance of innate processes for sleep maturation over time [21–23, 50]. Mothers of older infants in this study did not spend significantly more or less time asleep.

Evolved mammalian behaviour involves potential maternal sleep behavioural and endocrinal adaptive mechanisms postnatally [26, 50]. As well, sleep of new mothers and infants is shaped by cultural and social influences on daily activity patterns and our study is novel in examining these. Such influences include cultural or gender norms about infant sleep [20], and women’s leisure time, or affecting family members’ expectations about maternal employment or unpaid work, and gendered allocations of responsibilities for earning income or caring for young children.

Our study highlights that because infants require feeding and care at night, new mothers experience substantially lower and disrupted sleep hours compared to norms evident in time use research for adult populations, where sleep is categorised as a ‘necessary’ activity. A recent OECD study of populations aged 15–64 found that personal care, including sleeping and eating, accounts on average for 46% (11 h) of a 24-h day or around 77 h a week [51]. The remaining time is spent on leisure (20% of people’s total time, being 5 h a day, or around 34 h a week), and in employment or study (on average 19% of people’s time). This raises the question of whether new mothers attempting conformity to unrealistic social norms for adult sleep may induce changes in feeding practice away from exclusive breastfeeding, where prevailing social norms are inconsistent with biological norms for mother-infant dyads. By contrast, new mothers’ personal time was shown in this study to be well below 70 h a week. Mothers’ free time during the post-natal months was also low by comparison at around 20 h a week, again raising the question about how maternal expectations about leisure may or may not adapt to social norms.

The usual focus regarding infant care and feeding is on child health and development. Time is increasingly recognised as a resource needed to enable parents to provide the nurturing care underpinning optimal early child development. The recent *Lancet* series on child development cited policies including parental leave and breastfeeding breaks as key elements of evidence based approaches to optimising early childhood development [52].

Access to time resources also has implications for women’s health and well-being. Results from this study bring into focus the demands on women’s time to meet their own needs for sleep and leisure when they are caring for an infant, regardless of feeding method. Interestingly, in this study the unpaid work hours of new mothers differed significantly by feeding group but this interacted with infant age. Among breastfeeding mothers the introduction of solids seems to increase unpaid work hours as the infant got older, whereas in formula feeding mothers, unpaid work hours reduced with infant age but remained much higher than for breastfeeding mothers at 9 months. Relatedly, in previous research [28], maternal employment hours also interacted statistically with infant age and whether or not the mother was breastfeeding. Help received from other family members with infant care also did not differ by feeding group, and was minimal for most mothers.

Such complex time use relationships point to potential importance of paid or unpaid work commitments in constraining women’s capacity to adapt or adjust to reduced sleep or leisure opportunities after the birth of an infant. Together, these also suggest that gender equitable strategies for adjusting to night-time parenting involves reducing maternal work (employment obligations or housework) commitments, not only sharing infant care or feeding or altering feeding regimes. At the time of the TUSNM, only around 40% of new mothers took paid maternity leave, and this averaged less than 3 months in duration [53].

### Limitations

Our study has several limitations. It addressed maternal time use including sleep, but did not measure outcomes, or perceptions and expectations about sleep. Its generalisability to different populations may be limited by self-selection bias, as participation was voluntary. The small sample size limits the conclusions that can be drawn about the statistical significance of comparisons between particular feeding subgroups. Sensitivity analyses of results in Table 4 show that alternative statistical analysis strategies of removing the non-breastfeeding groups (exclusive formula feeding (B) and formula milk plus solids (E)) or alternatively combining (groups exclusive formula feeding (B) and mixed breast milk and formula no solids (C)) did not alter our findings regarding statistically significant variables or effects of feeding group or infant age on predicted means. Findings regarding maternal time use may not be generalizable to other countries. Although study participants had broadly homogenous socio-demographic characteristics, there is potential confounding from missing variables, due to mothers self-selecting into breastfeeding based on a complex combination of personal and social characteristics. These may include mental health variables such as anxiety or depression, or the availability of social and health services support, including for example paid maternity leave [54]. Higher representation of better educated, breastfeeding women in our sample may bias the results if the sample differs markedly from the population at large (such as in their degree of commitment to breastfeeding notwithstanding potential sleep loss) in how infant feeding and maternal sleep are linked. Reverse causation is also possible for the associations identified – the amount of time mothers allocate to personal care activities including sleeping could determine their likelihood of maintaining exclusive or partial breastfeeding, rather than feeding practice determining maternal time use such as that utilised for maternal sleep.

Future studies could explore whether endocrine factors may be implicated in different patterns of maternal time use between mothers practicing different degrees of breastfeeding.

Future research could also explore how equally the hours of work (paid and unpaid) are distributed between parents in the year after birth of an infant, how this is affected by paid maternity leave access and duration, and whether paternity leave increases gender equality in unpaid domestic work, as distinct from childcare activities. The extent to which access to sleep and leisure time is socially patterned among new mothers is also an important area for investigation in larger, population-based samples of families with children. The introduction of the paid parental leave (PPL) scheme in Australia in 2011 and the forthcoming ABS TUS in 2020–21 provides the opportunity for such research.

## Conclusions

New mothers experience reduced sleep hours, more night-time parenting, and reduced time for personal care, leisure and recreation activities compared to adult norms, regardless of feeding method. The amount of time taken to care for infants even older infants is substantial, and is likely to be intense. Where mothers experience less sleep hours, they may reallocate time from less preferred activities such as paid employment and unpaid domestic work to gain necessary personal and free time. Flexibility to reallocate time this way as well as providing time for nurturing care and breastfeeding of the infant may also depend importantly on sharing paid or domestic work responsibilities with others.

Recognising the temporal as well as the physiological mechanisms by which maternal mental health and child development benefit from policies and interventions such as paid maternity leave may have important implications for the design and effectiveness of parental leave and other social policies in promoting nurturing care and feeding of infants. It may also inform practice and program development by those supporting families with infants and young children.

## Supplementary Information

**Additional file 1: Table S2.** Maternal weekly hours spent in unpaid childcare and other domestic work, personal care and free time activities, by no or any breastfeeding and by age of target infant^a)^

**Additional file 2: Table S3.** Maternal weekly hours spent in unpaid childcare, personal care and free time activities, by detailed feeding group and by age of target infant^a)^.

**Additional file 3: Table S4.** Maternal weekly hours spent in other unpaid work^a)^.

## Data Availability

The datasets used and/or analysed during the current study are available from the corresponding author on reasonable request.

## Abbreviations

ABS: Australian Bureau of Statistics
ANU: Australian National University
ANOVA: Analysis of variance
REML: Residual maximum likelihood
TUS: Time Use Survey
TUSNM: Time Use Survey of New Mothers
