# Supplementary material for: Association between breastfeeding and new mothers’ sleep: a unique Australian time use study
Source: Int Breastfeed J. 2021 Jan 6;16:7. doi: 10.1186/s13006-020-00347-z (PMC7788894; doi:10.1186/s13006-020-00347-z)
Supplement: Supplementary file 1 — Additional file 1: Table S2. Maternal weekly hours spent in unpaid childcare and other domestic work, personal care and free time activities, by no or any breastfeeding and by age of target infanta) [file 13006_2020_347_MOESM1_ESM.docx]

**Supplementary Table 2 Maternal weekly hours spent in unpaid childcare and other domestic work, personal care and free time activities, by no or any breastfeeding and by age of target infant^a)^**

| **A. Maternal weekly hours spent in activity, by no or any breastfeeding (for all target infant ages)** | | | | | |
| --- | --- | --- | --- | --- | --- |
| **Activity/Breastfeeding status** | **No breastfeeding**  (obs=27) | **Breastfeeding**  (obs=300) | | **Av. SED** | **p-value** |
| **Necessary time** |  |  | |  |  |
| Sleep, adult meals and other personal care time | 8.09 (65.48) | 7.96 (63.43) | | .15 | .409 |
| Maternal sleep | 7.62 (58.13) | 7.38 (57.41) | | .14 | .085 |
| **Committed time** |  |  |  |  |  |
| Childcare | 6.28 (39.39) | 6.92 (47.89) | | .24 | .007 |
| Sleeplessness | 1.28 (1.63) | 1.87 (3.48) | | .27 | .029 |
| Other unpaid work | 4.64 (21.51) | 4.21 (17.72) | | .21 | .039 |
| **Free time** | 4.72 (22.26) | 4.41 (19.47) | | .29 | .286 |
| Night-time (sleep plus disturbed sleep) | 7.82 (61.20) | 7.70 (59.32) | | .14 | .379 |
| **B. Maternal weekly hours spent in activity, by age of target infant (for no or any breastfeeding)** | | | | | |
| **Activity/Age of target infant** | **3 months**  (obs=86) | **6 months**  (obs=134) | **9 months**  (obs=107) | **Av. SED**^(b)^ | **p-value** |
| **Necessary time** |  |  |  |  |  |
| Sleep, adult meals and other personal care time | 8.01 (64.18) | 8.10 (65.59) | 7.97 (63.58) | .08 | .186 |
| Sleep | 7.47 (55.79) | 7.57 (57.32) | 7.46 (55.65) | .74 | .202 |
| **Committed time** |  |  |  |  |  |
| Childcare | 6.86 (47.1) | 6.56 (42.97) | 6.38 (40.66) | .11 | <0.001 |
| Sleeplessness | 1.73 (2.98) | 1.53 (2.33) | 1.46 (2.18) | .13 | .135 |
| Other unpaid work | 4.22 (17.77) | 4.51 (20.31) | 4.55 (20.70) | .10 | .003 |
| **Free time** | 4.65 (21.60) | 4.46 (19.86) | 4.59 (21.09) | .14 | .342 |
| Night-time (sleep plus disturbed sleep) | 7.76 (60.25) | 7.83 (61.28) | 7.70 (59.24) | .07 | .154 |

**^a)^**Residual maximum likelihood analysis of maternal weekly hours spent in unpaid childcare, personal care and free time activities, by detailed feeding group and by age of target infant, using linear mixed model. Predicted means with back-transformed means in parentheses. Note that back-transformed means will be similar, but not be the same as the means for the original data, due to the transformation and the unbalanced nature of the data. ^(b)^ Av. SED is average standard error of difference.
