# Supplementary material for: Association between breastfeeding and new mothers’ sleep: a unique Australian time use study
Source: Int Breastfeed J. 2021 Jan 6;16:7. doi: 10.1186/s13006-020-00347-z (PMC7788894; doi:10.1186/s13006-020-00347-z)
Supplement: Supplementary file 3 — Additional file 3: Table S4. Maternal weekly hours spent in other unpaid worka). [file 13006_2020_347_MOESM3_ESM.docx]

**Supplementary Table 4: Maternal weekly hours spent in other unpaid work^a)^**

| **Interaction between age of target infant and detailed feeding group (P=.039, Av. SED=.64)** | | | |
| --- | --- | --- | --- |
| **Feeding group** | 3 months | 6 months | 9 months |
| A. Breastfed only | 4.00 (16.02) | 4.10 (16.84) | 3.675 (13.51) |
| B. Formula only | 4.55 (20.69) |  | 3.811 (14.52) |
| C. Breastfed & formula fed | 4.01 (16.08) | 4.84 (23.46) |  |
| D. Breastfed & solids | 3.10 (9.59) | 4.23 (17.88) | 4.388 (19.26) |
| E. Formula fed &solids |  | 5.10 (25.96) | 4.746 (22.53) |
| F. Breastfed & formula fed & solids |  | 5.10 (26.05) | 4.133 (17.08) |

^a)^Residual maximum likelihood analysis of maternal weekly hours spent in unpaid childcare, personal care and free time activities, by detailed feeding group and by age of target infant, using linear mixed model. Predicted means with back-transformed means in parentheses. Note that back-transformed means will be similar, but not be the same as the means for the original data, due to the transformation and the unbalanced nature of the data. ^(b)^ Av. SED is average standard error of difference.
